# A longitudinal study of the development of the saliva microbiome in infants 2 days to 5 years compared to the microbiome in adolescents

**DOI:** 10.1038/s41598-020-66658-7

**Published:** 2020-06-15

**Authors:** Pernilla Lif Holgerson, Anders Esberg, Andreas Sjödin, Christina E. West, Ingegerd Johansson

**Affiliations:** 1 Department of Odontology, Section of Pediatric Dentistry, Umeå University, SE-90187 Umeå, Sweden; 2 Department of Odontology, Section of Cariology, Umeå University, SE-901 87 Umeå, Sweden; 3 Division of CBRN Defence and Security, Swedish Defence Research Agency, SE-906 21 Umeå, Sweden; 4 Department of Clinical Sciences, Pediatrics, Umeå University, SE-90187 Umeå, Sweden

**Keywords:** Microbiology, Microbial communities, Microbiome, Paediatric dentistry

## Abstract

Understanding oral microbiota programming attracts increasing interest due to its importance for oral health and potential associations with systemic diseases. Here the oral microbiota was longitudinally characterized in children from 2 days (n = 206) to 5 years of age and in young adults (n = 175) by sequencing of the v3-v4 region of the 16S rRNA gene from saliva extracted DNA. Alpha diversity increased by age, with 2-day- and 3-month-old infants in one sub-group, and 18-month- and 3-year-old children in another. *Firmicutes* decreased up to 3 years of age, whereas *Proteobacteria, Actinobacteria*, *Bacteroidetes* and *Fusobacteria* abundances increased. *Abiotrophia*, *Actinomyces, Capnocytophaga, Corynebacterium*, *Fusobacterium*, *Kingella, Leptotrichia, Neisseria* and *Porphyromonas* appeared from 18-months of age. This was paralleled by expansions in the core microbiome that continued up to adulthood. The age-related microbiota transformation was paralleled by functional alterations, e.g., changed metabolic pathways that reflected e.g., breastfeeding and increasing proportions of anaerobic species. Oral microbiotas differed by feeding mode and weakly by mode of delivery, but not gender, pacifier use or cleaning method or probiotic intake. The study shows that the saliva microbiota is diverse 2 days after birth and under transformation up to 5 years of age and beyond, with fluctuations possibly reflecting age-related environmental influences.

## Introduction

Characterization of the oral microbiota has gained increasing interest following demonstration of its importance for oral health and potential associations with systemic diseases, such as autoimmune diseases, cancer and myocardial infarction^1–4^. However, the trajectory to a health- or disease-associated oral microbiome from birth to adulthood is largely uncharacterized, as is the role of childhood microbiota traits in health or disease later in life.

Bacteria that are characteristic of the mouth are found in the amniotic fluid^5^, but bacteria establishment in the mouth and other parts of the gastro-intestinal canal becomes significant from birth^6^. In the neonatal period, targeted characterization of the oral microbiota i.e., by culture and PCR, has revealed species in the *Staphylococcus* genus during the first two days of life^7,8^. Longitudinal maturation of the oral microbiota has been studied by culture^9,10^ and DNA-based methods^11–14^ with samplings beginning at 2–3 months of age and usually up to 12–24 months of age^14^. Some studies, have followed the children to 3 years and beyond, which extends characterization into an age with radically changed colonization conditions due to that all primary teeth have erupted^11–13^. Thus, in the first year of life a successive introduction of species in the *Streptococcus*, *Gemella, Granulicatella, Haemophilus* and *Rothia* genera is reported^15–17^, whereas species in the *Actinomyces, Porphyromonas, Abiotrophia* and *Neisseria* genera appear around one year of age and later^9–13^.

During the early phase of life, the oral microbiota is highly variable with, apart from host traits, main influences from family member transmission, cultural- and age-related food differences and introduction of the teeth. Some studies report that the oral microbiota stabilizes into subject- and niche-defined ecosystems at around 2 years of age^11,14,17,18^, but others find that equilibrium occurs later^19^. The optimal design to understand age-related maturation and stabilization of the oral microbiota and its determinants is a longitudinal study with closely repeated sampling. However, to date, the resolution, i.e., number of sampled ages, from the neonatal period to childhood is restricted to a few ages in each study, limiting a fine-tuned understanding of when and how the microbial community in the mouth obtains a unique taxonomical and functional trait profile.

The aim of the present study was to longitudinally follow ecological changes, including taxon profiles and functional potential of the saliva microbiota, from 2 days to 5 years of age and to compare these changes with taxon profiles of young adults. Additionally, microbiota diversity was evaluated in relation to a panel of environmental exposures, including mode of delivery and infant feeding, use of a pacifier and probiotic products.

## Results

### Study group characteristics

At baseline, 210 caregivers consented to participate with their new-born baby, but due to insufficient sample collection or recent antibiotic exposure, only 206 infants were included. A flow chart for the children is shown in the Supplementary Fig. S1. All infants were healthy and had a birth weight above 2 500 grams. Due to family relocations or lack of time, the number of participating children decreased over time (see Supplementary Fig. S1). Additionally, 18 children had antibiotic use within 3 months before sampling and were excluded (Table 1). In total, 78 children participated at all sampling occasions, but 7 of these had antibiotics within 3 months before sampling, leaving 71 children for strict longitudinal analyses. 176 young adults consented to participate, but one subject was excluded due to failed sequencing. Summary characteristics of the included children and the 175 young adults are shown in Table 1 and additional information on participant inclusion is presented in the Supplementary information.

**Table 1:** Characteristics of the included children (2 days to 5 years of age) and of the included young adults (18 year of age).

	2 days	3 months	18 months	3 years	5 years	18 years
	n = 207^a^	n = 159	n = 141	n = 141	n = 119	n = 175
Boys, %	53.1	54.1	57.9	55.1	51.3	51.4
Age, mean (95% CI)	—	3.1 (2.8, 3.3)	18.0 (17.9, 18.3)	2.9 (2.7, 3.3)	5.0 (4.8, 5.2)	18.1 (18.0, 18.3)
Caesarean section, %	6.9	—	—	—	—	—
Breastfed^b^, %	98.5	82.4	5.0	2.0	0.0	—
Antibiotics^c^, n (%)	1 (0.5)	4 (2.5)	9 (6.5)	1 (0.7)	3 (2.5)	0 (0)
Probiotic drops, %	—	17.5	4.7	—	—	—
Use of pacifier, %	—	69.0	65.5	43.2	0.8	—
Daily tooth brushing^d^, %	—	—	—	96.5	99.1	80.0
Caries, %	—	—	—	8.8	10.0	69.7
defs/DeFS, mean (95% CI)	—	—	—	1.2 (1.1, 2.0)	2.7 (1.3, 5.0)	4.7 (3.7, 5.8)
Illumina analysis^e^, n	206	155	132	140	116	175

^a^Appropriate sampling was not possible for 3 infants.

^b^Exclusive or partial breast-feeding.

^c^Children receiving antibiotics within 3 months before sampling. For the 18-year-old subjects, antibiotic use within 6 months prior to sampling was an exclusion criterium.

^d^Daily brushing refers to brushing 1–2 times per day.

^e^Numbers after exclusion of participants that received antibiotics within 3 months prior to sampling.

### Overall sequencing results

In summary, 5 272 amplicon sequence variants (ASVs) with ≥98.5% identity with a reference sequence in the eHOMD and represented by ≥2 sequences were identified. Overall, 11 phyla, 129 genera and 484 named species or unnamed phylotypes were taxonomically assigned. Details of the overall sequencing results are described in the Supplementary information online.

### Microbiota profiles from 2 days to 18 years of age in cross-sectional comparisons

**Figure 1:**
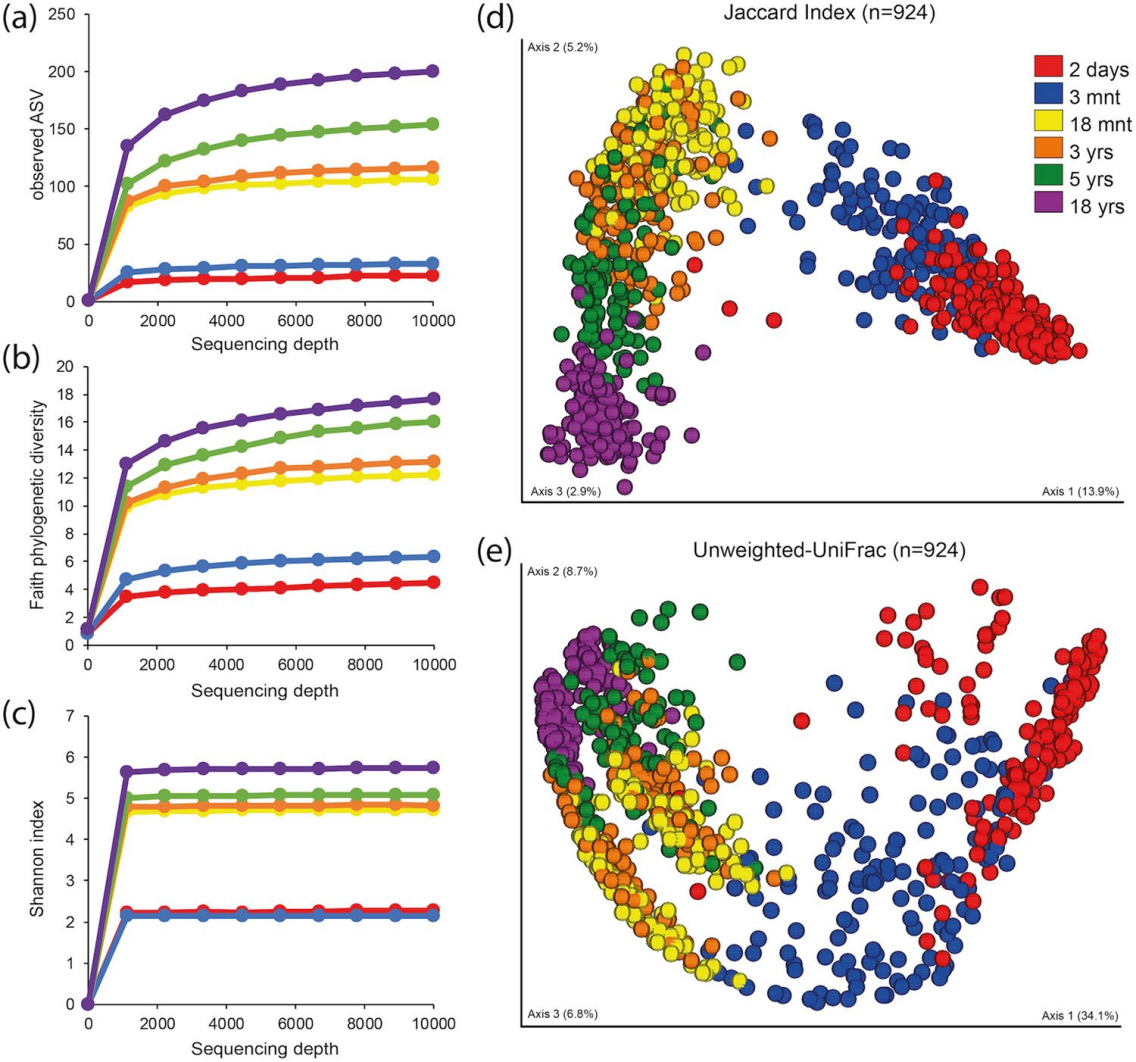
Saliva microbiota alpha- and beta diversity from 2-days to 18 years of age. Amplicon sequence variant (ASV) based diversities built on results from 924 saliva samples collected at ages 2-days, 3- and 18-months and 3-, 5- and 18-years. Figures a-c show rarefaction curves (alpha diversity) based on (**a**) observed amplicon sequence variants (ASVs), (**b**) Faith’s phylogenetic diversity and (**c**) Shannon index at varying sequencing depths. Figures (**d**,**e**) show PCoA plots illustrating beta-diversity based on (**d**) Jaccard diversity, and (**e**) Unweighted Unifrac distance for the same ASVs. The first component explained nearly 14% based on the Jaccard diversity of the individual variation and 34% based on the Unweighted Unifrac distance.

Alpha diversity, whether evaluated as observed ASVs (qualitative measure of community richness, Fig. 1a), Faith’s phylogenetic diversity (qualitative measure of community richness incorporating phylogenetic relationships, Fig. 1b), or the Shannon index (quantitative measure of community richness, Fig. 1c), increased with age but with 2-day- and 3-month-old infants clustering in one stratum and 18-month- and 3-year-old children in a second (Fig. 1a–c).

With the aim of characterizing ecological changes in the saliva microbiota by age, we selected qualitative data for ASV analyses of beta diversity. Thus, the Jaccard distance (Fig. 1d) and unweighted-UniFrac distance (including phylogenetic relationships, Fig. 1e) among the 924 samples tended to aggregate participants by age, although some individual overlap occurred, especially when the children were 18 months and 3 years old. Although no absolute separation was apparent between adjacent age groups, the overall difference was significant when tested with PERMANOVA (p < 0.001) and between age groups in post hoc follow-up comparisons with FDR corrected p-values < 0.001 for all comparisons.

**Figure 2:**
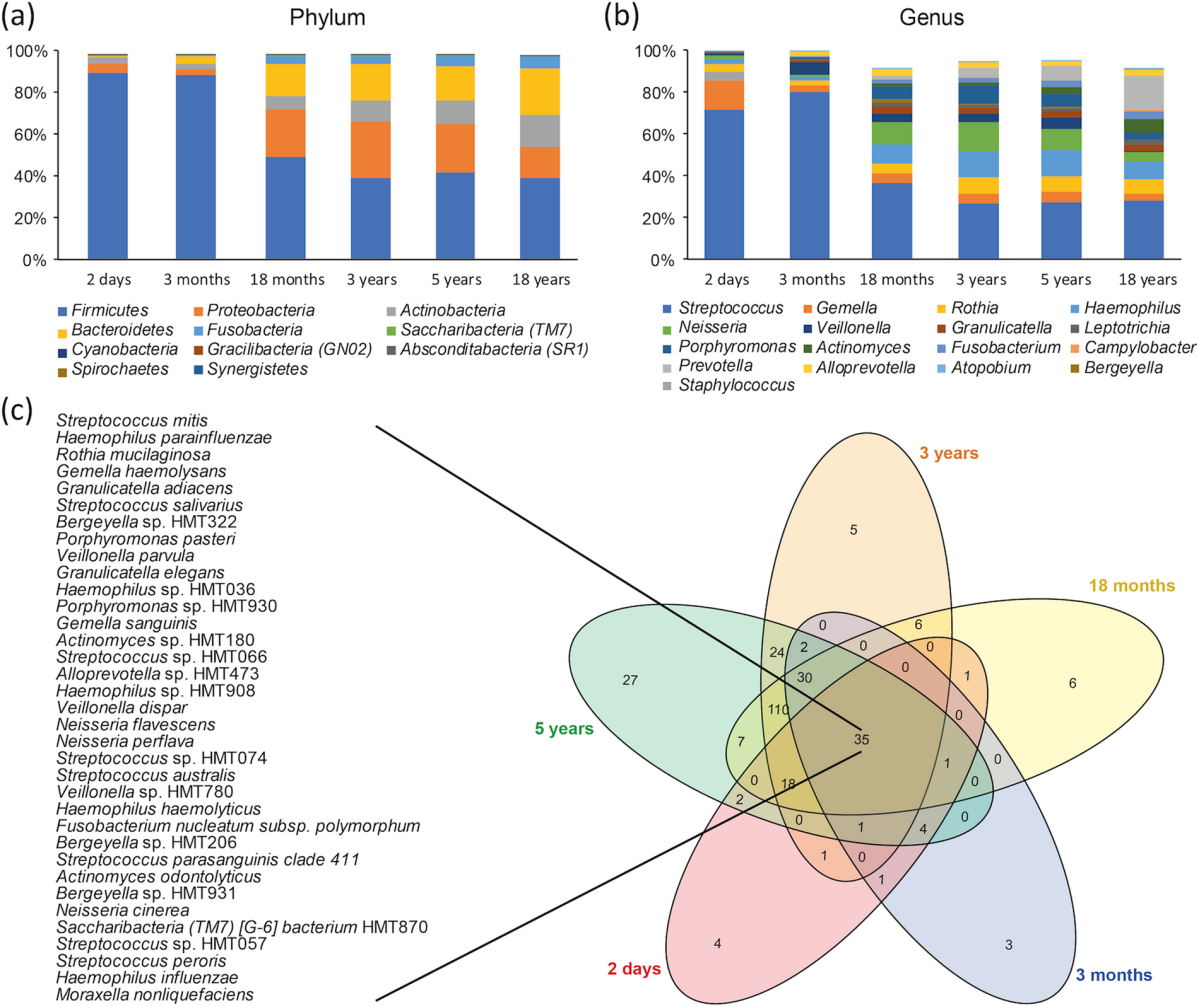
Saliva microbiota transformation from 2 days to 18 year of age. Taxa detection prevalence (% carriers) at the (**a**) phylum and (**b**) genus level according to the eHOMD database assignments at 98.5% identity and at least 2 ASVs per taxa for 924 saliva samples collected at the ages 2 days, 3 and 18-months and 3-, 5- and 18-years. The Venn diagram (c) illustrates taxa overlaps with an attached list of the 35 taxa identified at all sampling occasions, i.e., from 2 days to 5 years, in at least three children.

The detection profile of eHOMD-identified taxa decreased from approximately 80% to 40% in the *Firmicutes* phylum from 2 days to 18 months of age and onward (Fig. 2a). This decrease was explained mainly by a decrease in the relative abundance of the *Streptococcus* genus as well as in that of *Gemella* as a function of that the diversity increases over time (Fig. 2b). Corresponding increases occurred in the *Proteobacteria, Actinobacteria*, *Bacteroidetes* and *Fusobacteria* phyla and the introduction of species in 6 less prevalent phyla by increasing age (Fig. 2a). A Venn diagram, which was restricted to the 5 sampling occasions in children for readability reasons, showed that 35 species were found at all ages in at least 3 children (Fig. 2c). Age-stratified prevalence (% carriers) and relative abundance (%) of the 484 species present in 2 or more children/young adults are shown in the Supplementary Table S1. Among these species, only *Streptococcus mitis* and *Gemella haemolysans* were present in >95% and 3 more in >75% (3 in *Firmicutes* and 1 in *Actinobacteria*) when the infants were 2 days old (Supplementary Table S2). In addition, *Staphylococcus lugdunensis* and *Staphylococcus pasteuri* were present in up to 50% at this age. At 3 months, 7 species were present in >75% of the infants (6 in *Firmicutes* and 1 in *Actinobacteria*), which increased to 32 species at 18 months, 37 at 3 years, 55 at 5 years and to 75 species at 18 years (Supplementary Table S2).

**Figure 3:**
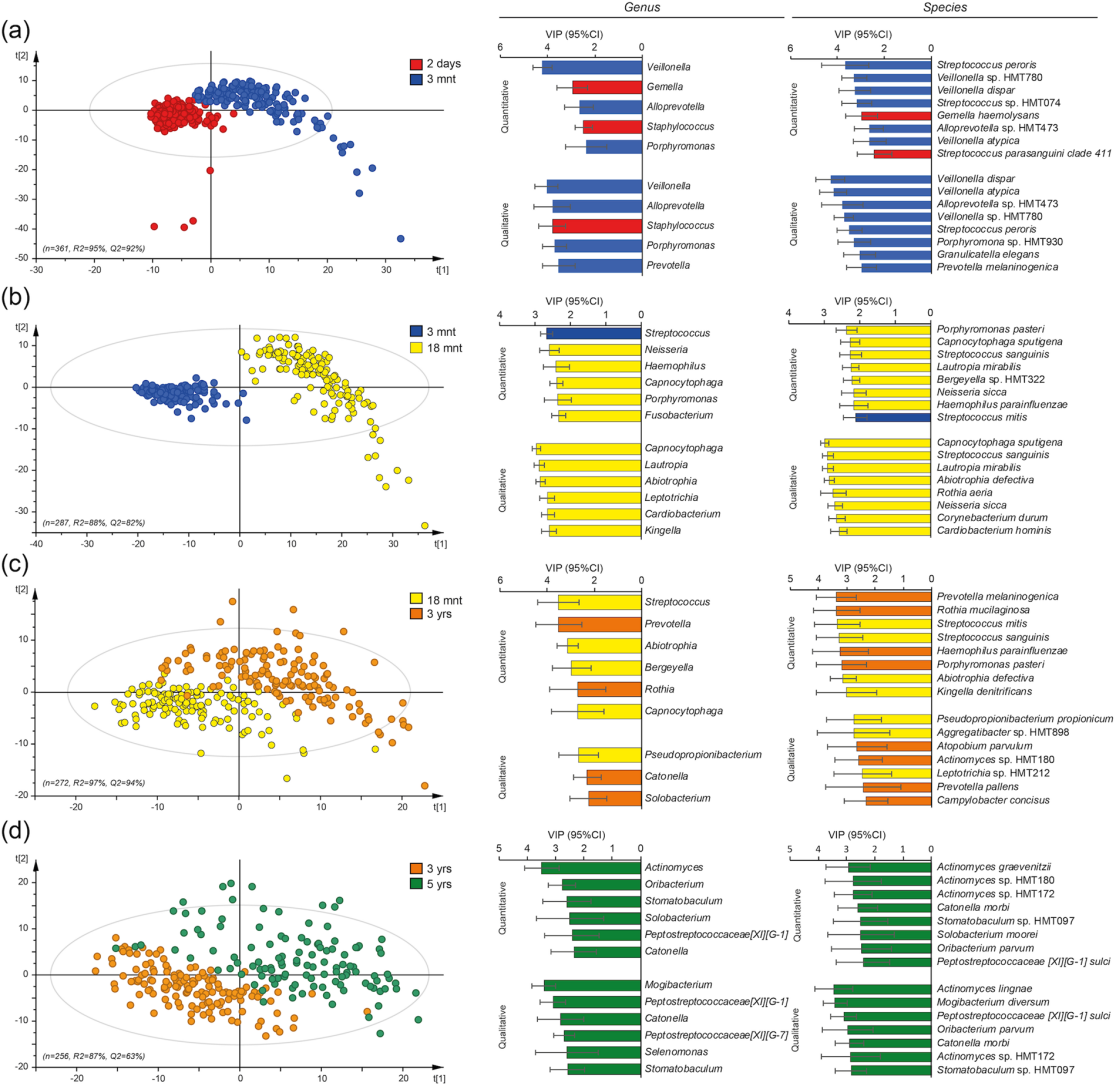
Saliva bacterial species driving age group separations in 2-day- to 5-year-old children. Pairwise comparisons of saliva-swab samples collected when the children were 2 days, 3- and 18-months, and 3- and 5-years of age (n = 749) were made in PLS regression models. The left panel shows PLS score plots with each dot representing a child when comparing (**a**) 2 days versus 3 months, (**b**) 3 months versus 18 months, (**c**) 18 months versus 3 years, and (**d**) 3 years versus 5 years old children. The scores plot scale results from the projection of the data onto the principal components, with t[1] and t[2] representing the new created variables summarizing the x-variables for component 1 and 2, respectively. The bar graphs to the right show the top genera and species, i.e., VIP values > 2.0, driving the separation of the individuals in each age group comparison.

As a next step, species that were influential for separating children by age based on their microbiota profiles were searched by multivariate partial least square (PLS) regression (Fig. 3a–d). PLS was chosen since it allows co-variation among bacterial taxa. The top influential genera for separating 2 day from 3 month old infants were *Staphylococcus* (relative abundance and presence (yes/no)) and *Gemella* (relative abundance); for separation between 3 and 18 month old children, the presence of *Capnocytophaga, Lautropia, Abiotrophia, Leptotrichia, Cardiobacterium*, and *Kingella;* between 18 month and 3 year old children, the presence of *Catonella* and *Solobacterium;* and between 3 and 5 year old children, the presence of *Mogibacterium, Peptostreptococceae[XI][G-1], Catonella, Peptostreptococceae[XI][G-7], Selenomonas* and *Stomatobaculum*. The full list of VIP-values from the PLS models is presented in the Supplementary Table S3. The PLS results were confirmed in LEfSe/LDA analyses, with the addition of strains in the *Lactobacillales* order which were associated with being 3 or 18 months old (Supplementary Fig. S2).

### Microbiota profile and environmental exposures

**Figure 4:**
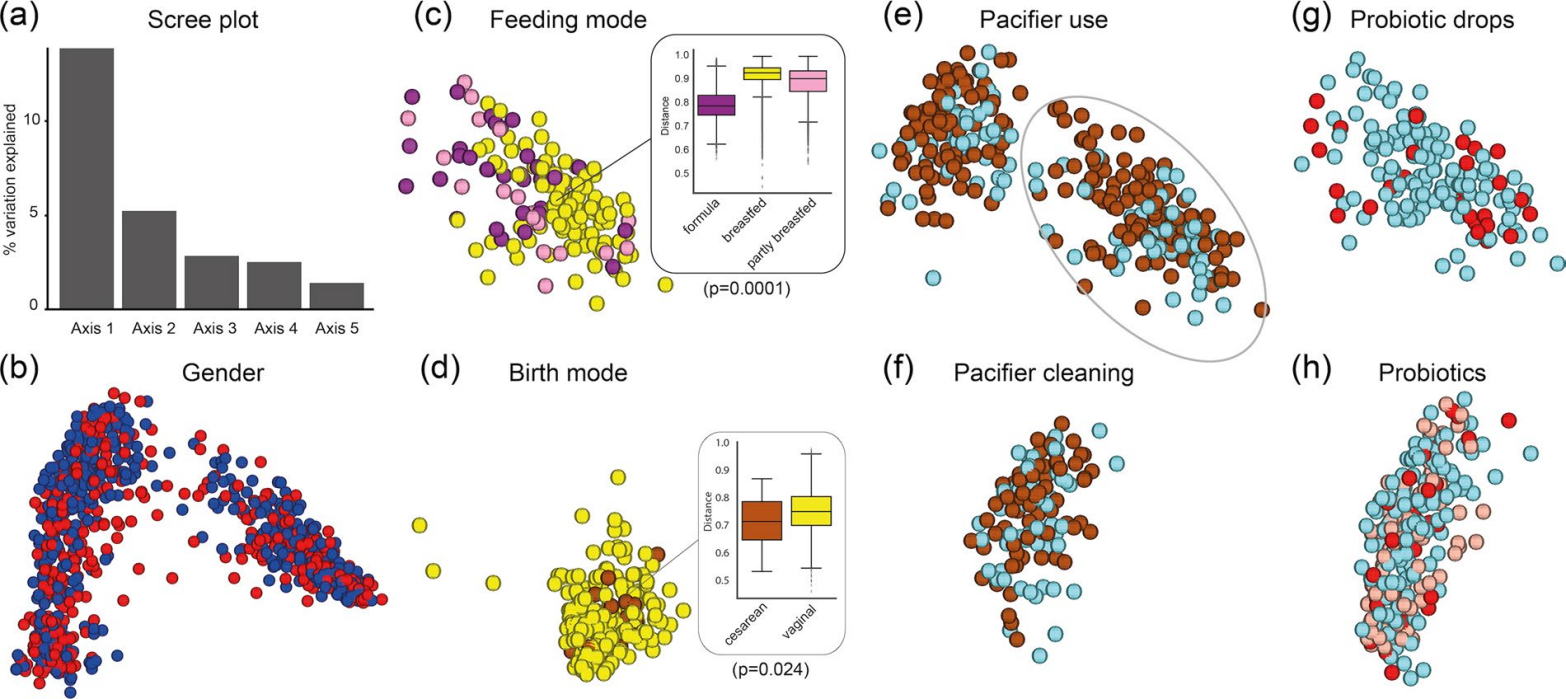
Saliva microbiota diversity by gender, feeding- and birth-mode, pacifier habits, and exposure to probiotic bacteria. Figure (**a**) shows the scree plot from the Principal Coordinates Analysis (PCoA) eigen-vector analysis of 924 saliva-sample-matrix, i.e. samples collected in 2 days to 18 year old participants. Figures b - h are the Jaccard PCoA plots for (**b**) gender, (**c**) feeding mode in 3-month-old infants, (**d**) birth mode in 2-days-old infants, (**e**) use of a pacifier or not versus in 3 months (encircled symbols) and 18 months old children, (**f**) parental cleaning mode of the pacifier if the child used it at 18 months of age (the question was introduced in the 18-month questionnaire), (**g**) use of probiotic drops in 3-month-old infants (red dots), and (**h**) use of other probiotic products in from 3- (red dots) and 5 year old children (pink dots).

The Jaccard index based multivariate (PCoA) model (Fig. 4a) was also evaluated in relation to a selection of external environmental and possibly confounding factors in. These factors included gender (Fig. 4b), mode of feeding at age 3 months (Fig. 4c), mode of delivery at 2 days of age (Fig. 4d), use of pacifier or not at ages 3 and 18 months (Fig. 4e) and cleaning procedure (in 18 month olds only since the question lacked earlier) (Fig. 4f), exposure to probiotic drops at age 3 months (Fig. 4g), and other types of probiotic products when the child was 3 or 5 years of age (Fig. 4h).

The microbiota profile did not differ between boys and girls, nor between those using a pacifier or not or if the parents cleaned it by sucking or rinsing it with water or if the child was given probiotic bacteria at 3 months of age or later. However, 3-month-old formula-fed infants separated from those being fully breastfed, with an overall statistically significant difference (PERMANOVA FDR p < 0.001) between the three feeding groups (fully or partially breastfed and formula fed), conferred by a difference between the fully breastfed (highest diversity) and formula-fed (lowest diversity) groups (Fig. 4c). Further, though not possible to see in the PCoA plot projection, PERMANOVA indicated a moderate statistically significant difference (p = 0.024) between 2-day old infants that had been delivered vaginally and those delivered by Caesarean section with a higher median Jaccard diversity index in the vaginally delivered infants (Fig. 4d).

### Longitudinal changes in the microbiota profile from 2 days to 5 years of age

**Figure 5:**
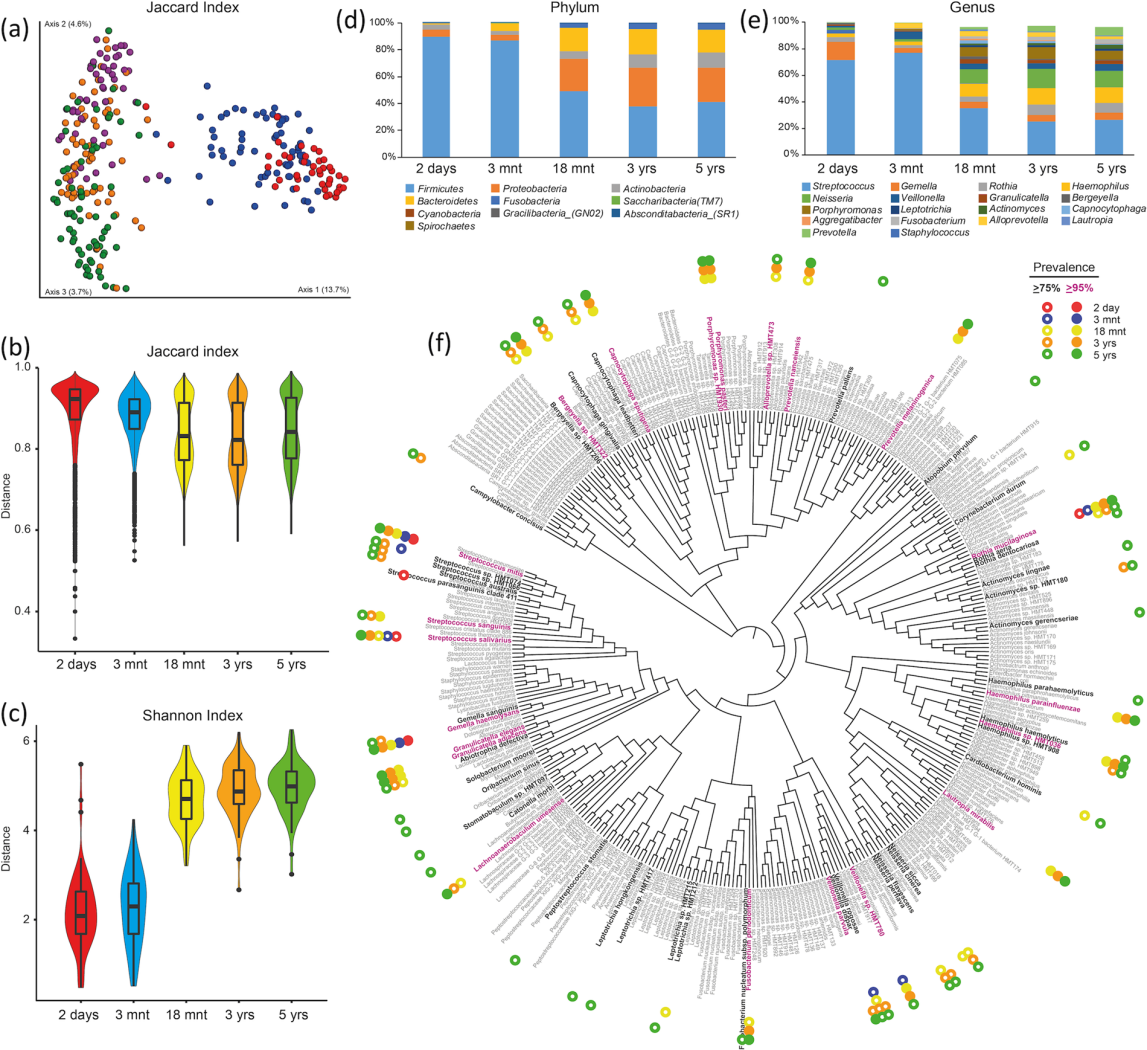
Saliva microbiome characterization for children with 5 year repeated samplings from birth to pre-school age. The results are based on 71 children with saliva swab samples collected when the children were 2-days, 3- and 18-months, and 3- and 5-years of age. None had any antibiotic exposure in the preceding 3-month period. Figure (**a**) shows the beta diversity from the Jaccard dissimilarity PCoA plot (PERMANOVA p < 0.001 among and between age groups). Figure (**b**,**c**) are violin plots including box plots for (**b**) the Jaccard beta-diversity differences, and (**c**) the Shannon index for alpha diversity. Figures (**d**,**e**) show taxa proportions at the phylum and genus level by age, and (**f**) the phylogenetic tree for bacterial species detected among the 71 children and with color-coded indications of taxa found in 75% (open circles) or 95% (filled circles) of the children at different ages.

Some characteristics for the subset of 71 children who were included in strict longitudinal analyses are presented in the Supplementary Table S4. A Jaccard PCoA plot of ASV-based beta diversities for these children largely followed that of the larger group of 749 children (Fig. 5a). Complementary violin plots of the Jaccard index showed slightly higher diversity scores and wider variations for the two younger age groups compared to those of the three older groups (Fig. 5b). For the alpha diversity, the median values for the Shannon index (within individual species diversity) increased continuously by age, with the largest increase between 3 months and 18 months. Additionally, the index range was widest for the youngest age groups compared to a more compact distribution around the median values at older ages, as displayed in the violin plot in Fig. 5c.

Phylum and genus profiles in the 71-child subsample were basically identical to those of all 749 children (Fig. 5d,e versus Fig. 2a,b), as was the pattern of taxa detected in 95% versus 75% of the infants (Fig. 5f).

**Figure 6:**
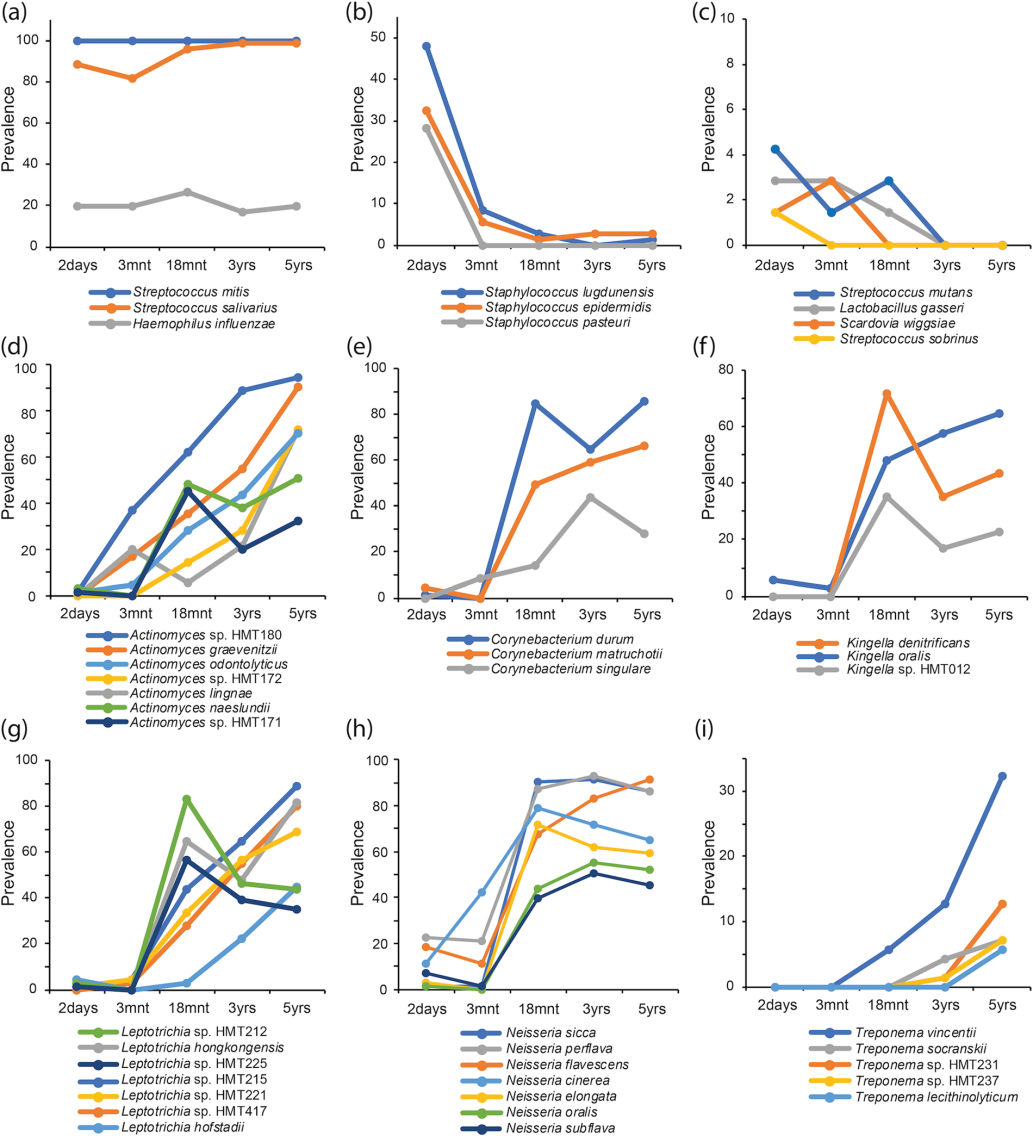
Longitudinal transformation of species detection in the saliva microbiota from birth to pre-school age. Scatter-line plots illustrating fluctuations of species prevalences, i.e., % of the 71 children with presence of the species in the saliva-swab sample, at the five repeated sampling occasions. Species presence was defined according to criteria described in the methods section. (**a**) the 3 bacterial species with virtually unchanged detection over the 5-year period; (**b,c**) species with decreasing detection; (**d–i**) a selection of species with increasing detection prevalence over the 5 year longitudinal study period.

The longitudinal fluctuations for a set of selected species are shown in Fig. 6a–i. The detection prevalence of 3 species (*Streptococcus mitis, Streptococcus salivarius* and *Haemophilus influenzae)* was maintained over the 5-year period (Fig. 6a), whereas that of 3 *Staphylococcus* species decreased (Fig. 6b) together with some low-frequency species previously associated with breastfeeding^20^ and caries^21,22^ (Fig. 6c). In line with the described changes in phylum and genus patterns, a large number of species increased with age, with some top species shown in Fig. 6d–i.

### Predicted functional potential from the 16S rRNA gene information

**Figure 7:**
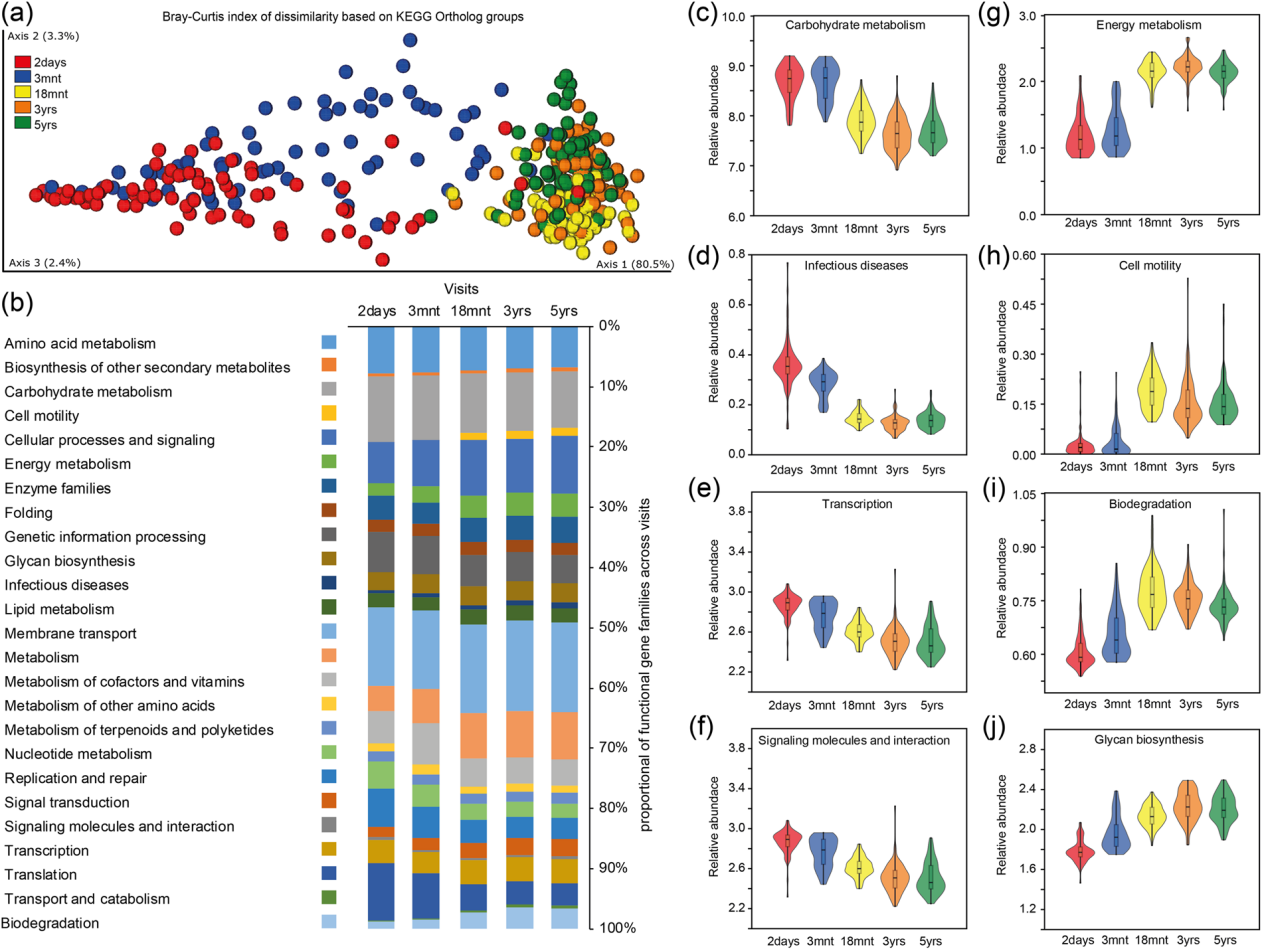
Predicted functional potential from the 16S rRNA gene information. By using Phylogenetic Investigation of Communities by Reconstruction of Unobserved States (PICRUSt2) within QIIME2, the identified 16S rRNA genomes were linked to pathways by ortholog annotation (KEGG orthologues, KO) to evaluate transformations in estimated functions in the saliva microbiome of 71 children with saliva swab samples from all five visits and free from antibiotics in the preceding 3-month period. (**a**) Bray-Curtis dissimilarity PCoA plot of participants based on their predicted abundance of KO orthologues (overall PERMANOVA, FDR, p = 0.0001 and between-age-group PERMANOVA, FDR, p < 0.009). (**b**) Proportions of major estimated functions at each age group. (**c**–**j**) Violin plots including box plots in a selected panel of functions illustrating changes between the five monitored ages (for all functions, overall PERMANOVA, FDR, p < 0.001).

By linking the ASV genomes to pathways by orthologue annotation (KEGG orthologues, KOs), differences in estimated bacterial functional capabilities in the 71 children with 5 repeated samples and free from antibiotics in the preceding 3-month period were compared using QIIME2 and Phylogenetic Investigation of Communities by Reconstruction of Unobserved States (PICRUSt2). The number of core KOs, i.e., those present in all subjects at a specific time point, increased over time from 1 503 estimated functions at 2 days to 4 774 at 5 years, with the greatest expansion (2 028 KOs) between 3 and 18 months. The PCoA plot based on the predicted abundance of KOs clearly separated the 2 day and 3-month age groups, but although the 18-month age groups tended to separate from the 5-year-old children, there was considerable overlap among the three older age groups (Fig. 7a). Nevertheless, the overall difference was statistically significant (PERMANOVA, FDR, p < 0.0001), and also in post hoc tests between age groups (PERMANOVA, FDR, p < 0.009). Qualitative prevalence (yes or no) and relative proportion of estimated KO functions (level 2) at each time point varied over time. The relative proportions of functions associated with amino acid and carbohydrate metabolism, cellular processes/singling, enzymes, folding, glycan biosynthesis, lipid metabolism and membrane transport, metabolism of cofactors, other amino acids and terpenoids and polyketides, as well as signalling molecules/interactions and transcription, appeared to be constant from 2 days to 5 years. However, the functions associated with cell motility, xenobiotic biodegradation, and catabolism increased, whereas the functions associated with genetic processing, nucleotide metabolism, and translation decreased from 2 days to 5 years (Fig. 7b). Furthermore, the functions associated with carbohydrate metabolism, infectious disease, transcription, and signalling molecules/interactions decreased after 3 months (Fig. 7c–f), whereas those associated with energy metabolism, cell motility, xenobiotic biodegradation and glycan biosynthesis increased over time (Fig. 7g–j) (overall, PERMANOVA, FDR, p < 0.0001). Some level 3 KO functions, such as taurine, hypotaurine and galactose metabolism, bacterial epithelial cell invasion and *Staphylococcus aureus* infection, decreased over time, whereas functions linked to energy metabolism, lipopolysaccharide biosynthesis proteins, pyruvate and methane metabolism, and membrane and intracellular structural molecules were the top candidates that increased over time. These data are found in the Supplementary Table S5.

## Discussion

In the present study, the saliva microbiota from birth to preschool was followed and compared with that of a group of young adults to identify trajectories in its transformation and stabilization as a basis for future studies on long-term associations with oral and general diseases. It is the first study that also evaluates potential functional transformation of the oral microbiota in the same age span. A continuous age-related increase in the species richness and the number of core KEGG orthologues and a statistically significant age-related difference in bacterial profiles (beta diversity) were found. Specifically, noteworthy findings are that the most distinct evolution in these measures took place between 3 and 18 months and that heterogeneity between participants increased by age. Furthermore, the impact of breast-feeding on the oral microbiota was confirmed, with potential associations with inferred bacterial functions.

The main purpose of the present study was to characterize the oral microbiota and predicted functions by an untargeted DNA-based method in a longitudinal setting with tighter resolution than that provided in earlier studies. Compared to that of the gut microbiota^23,24^, the maturation and plasticity of the oral microbiota have been sparsely studied in longitudinal settings using such methods^11–14^. To date, the largest longitudinal studies, in addition to the present study, are by Dzidic *et al*. (n = 90)^12^, Kahharova *et al*. (n = 119)^13^, and Kennedy *et al*. (n = 66)^14^. In concert, the present study and those three studies confirmed that saliva species richness increases with age and that the compositional profile tend to change with age. The present study demonstrates that the major increase in the number of different species in saliva occurs between 3 and 18 months, and combining our results with those from Kennedy *et al*.^14^ the most active age window may be narrowed to between 6 and 18 months, and with a slower incline thereafter. Age reflects time window-related events, such as mode of delivery and early feeding, use of a pacifier, and maturation of salivary glands and oral tissues, and a major event that occurs between 6 and 18 months is the introduction of a new colonization niche, i.e., non-shedding, negatively charged tooth surfaces. The overall age-related differences in microbial composition were striking, but of equal importance was that individuality in the microbiota transformation had already materialized in the first months of life. It may even be hypothesized that the earliest interactions between genetically regulated host traits and environmental factors are a continuum of intrauterine manifestations. These diverse situations and potential interactions, combined with accessibility, renders the mouth a unique environment and a multifaceted model to study the induction of microbiota transformation.

The present study also evaluated the association between the saliva microbiota and mode of delivery and early infant feeding. These two exposures have been extensively evaluated in relation to the gut microbiota^25,26^ and that of the mouth^14,27,28^ but with varying methodologies and findings. Here, the oral microbiota diversity of fully breastfed infants was significantly different from that of formula-fed infants, but separation was not distinct between full and partial breastfeeding, which may reflect a reporting bias but also individual traits in the child and maternal milk. Human milk is a continuous source of bacteria to the child´s mouth and of bacterial-metabolism- and attachment-regulating elements^29^. The determinants of the bacterial profile of human milk are not fully understood but are suggested to be under complex influences of factors associated with lactating women per se as well as retrograde inoculation from the child´s mouth^30^. Though beyond the scope of this paper, further examinations of the bacterial profiles combined with bioactive components may contribute to the understanding of the effect of human milk on oral microbiota establishment and associations with health outcomes, such as allergy and ear and upper airway infections^31–33^. The difference in saliva-swab microbiota diversity between 2-day-old infants delivered by the normal route or by Caesarean section was statistically significant by PERMANOVA but weak. The finding is in line with some studies but not others^8,14,34–36^ targeting the mouth, and the effects of Caesarean section delivery on the acquisition and development of the gut microbiota during the neonatal period or early infancy have been partly controversial^36,37^. Scandinavian children born by Caesarean section are reported to tend to have higher caries prevalence, which could support the hypothesis of a differing microbiota according to delivery mode, but potential confounding factors from other caries-associated factors are unclear^38,39^. Beyond the bacterial microbiota studied here, the oral cavity harbours various fungal species. Maturation of the oral mycobiome was recently reviewed by Azevedo *et al*.^40^ and found unrelated to breastfeeding but promoted by vaginal delivery. Collectively, it remains to elucidate the causality of the effects of Caesarean section on the oral microbiota and disease development and the role of confounding factors, including feeding mode at the time of sampling.

In the present cohort, oral microbiota profiles in 3 months to 3-year-old children did not differ between those who used a pacifier or not or whether the caregivers cleaned it by sucking or rinsing with water. This result is in line with Neves *et al*.^41^, who did not find any association between the use of a pacifier and polymerase chain reaction denaturing gradient gel electrophoresis (PCR-DGGE)-characterized oral microbiota, but is in contrast to Hesselmar *et al*.^42^, who reported that the restriction fragment length polymorphism (T-RFLP)-defined oral bacterial profile differed between 4 month old children of parents who cleaned the child´s pacifier by sucking or not. However, the methods for taxon determination differ between the latter and the present study, and a strict age comparison could not be performed because information on the cleaning procedure was not available when “our” children were 3 months old. These factors together with varying impacts of potential confounders may have contributed to the deviating results.

One unforeseen finding was the detection and subsiding of lactobacilli and mutans streptococci in early infancy. For the lactobacilli, we suggest two potential explanations based on (i) our previous report on lactobacilli (mainly *Lactobacillus gasseri*) during breastfeeding^20^ and (ii) that the lactobacilli reflect the use of probiotic drops at approximately 3 months of age to ease infant colic^43^. Studies show that probiotic bacteria remain only as long as they are administered^44^, which would match the reported intake of probiotic drops and detection of lactobacilli in the saliva swabs. Anyhow, no association was seen between self-reported intake of probiotic products and overall saliva microbiota diversity. For the mutans streptococci, previous studies have reported presence of *Streptococcus mutans* in approximately 30% at 3 and 6 months of age^45^. Here, the detection prevalence was considerably lower which may reflect differences in study population characteristics as well as detection methods. Based on our finding that the prevalence was highest already at 2 days of age and then subsided combined with that *S. mutans* preferentially colonize saliva-coated tooth surfaces, we suggest that *S. mutans* is a transient species at this very early age with the source being close oral contact with caregivers, i.e., kissing and licking the pacifier, which is in line with other previous reports in edentulous infants^46^.

The assessment of potential functions of the maturing microbiota revealed age-related transformations that paralleled the increasing complexity of the oral ecosystems. Hence, functions needed to maintain an operational microbiota in periods of homogenous feeding in infancy, as well as in exposure cascades later in life, were apparent. For example, bacterial functions linked to taurine and galactose metabolism, which are metabolites associated with breast milk or degradation of lactose in breast milk^47,48^ decreased with age, which might correlate with a shift in bacterial composition linked to the termination of breast-feeding. Other examples are the large expansion of microbiota functionality (+2 028 KOs) around the age when the first primary teeth appear compared to that later when intra-oral changes are less radical, e.g., 227 KOs between 3 and 5 years^28^, and the increasing citrate functions paralleling the introduction of bacterial taxa adapted to aerobic conditions^49^. Notably, predicted functions did separate the age groups, but separation was more distinct by the ASVs per se, which suggests enrichment of species with overlapping functions over time. However, inferences based on an approximately 400 bp 16S rRNA gene segment as a “marker gene” have limited ability to distinguish species and even less ability to delineate strains, due to high similarity in the targeted variable region of the 16S rRNA gene. Thus, the results should be interpreted conservatively due to the limitation of detecting intra-species variations.

The strengths of the present study relate to its longitudinal design starting close to birth, inclusion of healthy, normal weight infants only and exclusion of those taking antibiotics within three months before sampling, and that the resolution is tighter than that in previous studies, and that it adds functional transformation aspects to the oral microbiota in children. However, some potential limitations may be considered when the results are evaluated. First, although the number of participants is contextually comparably large, it does not allow for sensitivity analyses in strata defined by potential confounder or interaction factors. Furthermore, the 18-year-old group represents a cross-sectional panel, but waiting for sampling of the children that were longitudinally followed would likely increase the loss further. Additionally, the saliva of the 18-year-old subjects was collected by expectoration of chewing-stimulated saliva, but this approach was not possible in the younger children. Both methods lead to a mixture of saliva and mucosal membrane-colonizing bacteria, but although it cannot be excluded that the proportions differ, it is unlikely that this methodological discrepancy explains the differences seen between 5- and 18-year-old subjects. Finally, the participating families were part of general childcare programmes (medical and dental) running in Sweden. Therefore, the results on the saliva microbiota from the later sampling ages may not be representative for countries where organized child medical and dental care is not available. Such ecological differences in the oral microbiota were recently shown when comparing Swedish and Romanian adolescents^50^.

The present study describes oral microbiota composition and transformation by age in healthy children, but at the same time, it reveals several research questions related to the apparent individuality in bacterial acquisition and retention that need to be addressed in future studies. Thus, both maturation and final homeostasis seem influenced by determinants other than those directly associated with age. Such determinants may be so-called upstream factors, such as socio-economic conditions and environmental and cultural factors (including breastfeeding), or downstream factors, such as host biology and genetics. We suggest studies on the impact of host biology and genetics as well as on the effect of human milk composition on the oral and gut microbiotas.

In conclusion, the present study indicated that the saliva microbiota is already significantly diverse 2 days after birth and under transformation until 5 years of age and beyond, which is paralleled by changing profiles of functional proteins in the microbiota. Future studies may disentangle the role of inborn host factors versus upstream factors for individual diversity and future health associations. The limited difference in the oral microbiota between 2 days and 3 months and between 18 months and 3 years, respectively, may be useful in the design of studies targeting prospective associations with disease.

## Methods

### Subjects and sample collection

Healthy infants were recruited via the maternity ward at Umeå University Hospital, Umeå, Sweden, in conjunction with a mandatory screening for phenylketonuria when the infant was 2 days old. For 210 infants, the caregivers consented to participate. Healthy young adults (n = 176) were recruited at a dentist’s office at their annual control visit. Participants with antibiotic use within at least 3 months before sampling were excluded.

Saliva swab samples were collected with sterile cotton swabs when the children were 2 days, 3 and 18 months, and 3 and 5 years old. The cotton swabs were swirled in buffer, removed, and stored at −80 °C until used. From the young adults, chewing stimulated saliva was collected into ice-chilled test tubes which were placed in a −80 °C freezer within 4 hours. Further description is found in the Supplementary information online. Demographic, diet, dental, and medical information was obtained by questionnaires and from medical records at each visit.

The study was approved by the Regional Ethical Review Board, at Umeå university, Sweden, Dnr: 2011-90-31 M and 2016-239-32 M, and followed the Declaration of Helsinki and the General Data Protection Regulation (GDPR). All parents and young adults were given written and verbal information before signing an informed consent to participate and agreeing to that the information could be used for research. The project is reported in accordance to Strengthening the Reporting of Observational Studies in Epidemiology (STROBE) guidelines for cohort studies.

### Bacterial 16Sr RNA gene sequencing

Genomic DNA was extracted using the GenElute Bacterial Genomic DNA Kit (Sigma-Aldrich, St. Louis, MO, USA) from 150 µL saliva swab buffer and 200 μL saliva aliquots, respectively. All samples were extracted by the same person. The samples were centrifuged for 5 minutes at 13,000 rpm, lysed in buffer with lysozyme and mutanolysin, treated with RNase and Proteinase K, and purified and washed. The quality of the extracted DNA was estimated using a NanoDrop 1000 Spectrophotometer (Thermo Fisher Scientific, Uppsala, Sweden) and the quantity by the Qubit 4 Fluorometer (Invitrogen, Thermo Fisher Scientific, Waltham, MA, USA). The same extraction protocol was applied to Milli-Q Ultrapure Water and a mixture of known bacterial species serving as negative and positive controls, respectively.

Bacterial 16S rRNA gene v3‐v4 amplicons were generated by PCR using KAPA hot start ready mix (KAPA HiFi HotStart ReadyMix (2×), United States Wilmington, MA, USA) and the PCR amplification program: 98 °C for 3 min; 30 cycles of; 94 °C for 20 s, 51 °C for 20 sec, 72 °C for 20 sec; followed by 10 min at 72 °C and 4 °C to finish. PCR amplifications were done using 341 F (ACGGGAGGCAGCAG) forward primer and the 806 R (GGACTACHVGGGTWTCTAAT) primers, that include a linker sequence, the 12 bp barcode, and the Illumina adaptor defined by Caporaso^51^. The PCR product size was checked on a 0.8% agarose gel and the DNA concentration estimated using a Qubit fluorometer (Invitrogen, Thermo Fisher Scientific, Waltham, MA, USA) and equimolar libraries were pooled and purified using AMPure XP beads according to manufactures recommendations (Beckman Coulter, Stockholm, Sweden). Purified pooled amplicons were adjusted to 4 nM, spiked with 5% PhiX (Illumina, Netherlands), denaturated and diluted as described by Illumina (https://support.illumina.com/content/dam/illumina-support/documents/documentation/system_documentation/miseq/miseq-system-guide-for-local-run-manager-15027617-05.pdf) before loaded onto MiSeq cartridges (Illumina, San Diego, CA, United States) at the Swedish Defence Research Agency research facility in Umeå, Sweden. Totally, 11 cartridges were used for the samples from the children and 2 cartridges for the samples from the young adults. The samples were arranged age wise on the cartridges. Sequencing failed for one saliva sample from the young adults and 103 samples from the children with <4,900 reads. These samples were rerun for improved yield.

### Bacterial 16S rRNA gene sequence analysis and taxa assigent

The 16Sr RNA gene sequences were demultiplexed using deML^52^. Pair-end reads were fused, and primers, ambiguous and chimeric sequences were removed using default settings in DADA2 run within QIIME2 with resolution of amplicon sequence variants (ASVs)^53,54^.

Taxonomy was assigned to the ASVs using the Human Oral Microbiome Database e*HOMD* (http://www.homd.org/)^55^. ASVs with a 98.5% identity to a named species or unnamed phylotype and with at least two reads were retained. For reasons of simplicity, taxa are referred to as species in the text regardless of whether they are named species, or unnamed phylotypes.

ASV multiple sequence alignment, masking of highly variable positions, generation of a phylogenetic tree with midpoint rooting and assessments of diversity indexes were performed in QIIME2.

### Prediction of functional potential from the 16S rRNA gene information

Obtained representative ASV of 16S rRNA genomes from the 71 children who had attended all five sampling occasions and had not antibiotic intake within 3 months before sampling were search for potential molecular functions of the saliva microbiota at different ages using Phylogenetic Investigation of Communities by Reconstruction of Unobserved States (PICRUSt2)^56^ and the KO Database of Molecular Functions by ortholog annotation (KEGG orthologues, KO, https://www.genome.jp/kegg/ko.html). The steps included (i) creating a closed reference feature table in QIIME2 using the Greengenes database version 13_5, (http://greengenes.lbl.gov)^57^. which is the version PICRUSt2 is trained against, (ii) qiime diversity core-metrics analysis in QIIME2, and (iii) export of pathway abundancies and the feature table for down-stream analyses. Bray-Curtis PCoA diversity plot based on the predicted abundance of KOs were used to visualise age related shift in global microbiota functions and generated using QIIME2. Specific functions were highlighted with Violin plots including a Box plot generated using PAST3. Overall differences or specific age group differences were evaluated using PERMANOVA (PAST3, 9999 permutations, Benjamini–Hochberg corrected p-values).

### Data handling and statistical analyses

Descriptive data were calculated as means with 95% confidence limits (CI) or proportions (%) and non-parametric tests were used for univariate analyses using SPSS version 25 (IBM Corporation, Armonk, NY, USA). Relative abundance of identified taxa was expressed as the number of reads in proportion (%) of all reads for the individual and prevalence (detection rate) as the number of individuals where a taxon was identified in proportion of the group (%).

Species that were shared between groups were identified in a Venn diagram using Interactive Venn, (http://www.interactivenn.net/)^58^. Presence of a species was defined as yes if the species was found in three or more participants. A circular phylogenetic tree was created based on the 16S rRNA eHOMD genomes from the 71 children with repeated analyses and using iTOL: Interactive Tree of Life (http://itol.embl.de/).

Multivariate analyses included non-parametric permutational multivariate analysis of variance (PERMANOVA) to evaluate differences in microbial profiles (beta-diversity) using R-vegan 2.5.5 (adonis) within QIIME2, and partial least square regressions (PLS) using SIMCA P+ version 15.0 (Sartrius Stedim Data Analytics AB, Malmö, Sweden) to identify taxa that were influential for separating subjects by age^59^. PLS identifies directions in a swarm of independent X-variables (here bacterial species as a categorical (present/not present) or continuous measure (relative abundance)) that characterize X well and are related to Y (here age). The software scales all variables to unit variance, and performs a K-fold cross-validation where 1/7^th^ of data are systematically kept out when fitting the model and predicted from the remaining data (Q^2^-values). The results were displayed in score loading plots where each symbol represents an observation. PLS also provides variable loadings for identification of influential taxa in the projection. The loadings are expressed as Variable In Projection (VIP with 95% CI) values and PLS correlation coefficients. VIP-values and PLS correlation coefficients where the measures of variation do not include zero are considered statistically significant.

Additionally, the linear discriminant analysis effect size (LEfSe) method, (https://huttenhower.sph.harvard.edu/galaxy)^60^ was used to identify taxa that differed in relative abundance between groups, i.e. age.

P-values were considered statistically significant at a False Discovery Rate FDR  <  0.05.

## Supplementary information

Supplementary information.

## Data Availability

Sequence data have been deposited to the European Nucleotide Archive (ENA) under accession number PRJEB35824. The ethical approval and GDPR apply restrictions to the availability of individual level data which are therefore not publicly available. Such data are however available from the authors upon reasonable request and with permission of the Swedish Ethical Review Authority.
